# Comparative analysis of long noncoding RNA and mRNA expression provides insights into adaptation to hypoxia in Tibetan sheep

**DOI:** 10.1038/s41598-022-08625-y

**Published:** 2022-04-21

**Authors:** Fan Wang, Jianbin Liu, Qiaoying Zeng, Deqing Zhuoga

**Affiliations:** 1 College of Veterinary Medicine, Gansu Agricultural University, Lanzhou, 730070 China; 2 China Agricultural Veterinary Biological Science and Technology Co., Ltd., Lanzhou, 730046 China; 3 Lanzhou Institute of Husbandry and Pharmaceutical Sciences, Chinese Academy of Agricultural Sciences, Lanzhou, 730050 China; 4 Institute of Livestock Research, Tibet Academy of Agriculture and Animal Science, Lhasa, 850000 China; 5 Sheep Breeding Engineering Technology Research Center, Chinese Academy of Agricultural Sciences, Lanzhou, 730050 China

**Keywords:** Genetics, Molecular biology, Physiology, Biotechnology, Functional genomics, Genomics

## Abstract

Tibetan sheep have lived on the Qinghai-Tibetan Plateau for thousands of years and have good adaptability to the hypoxic environment and strong disease resistance. However, the molecular mechanism by which Tibetan sheep adapt to this extreme environment, especially the role of genetic regulation, is still unknown. Emerging evidence suggests that long noncoding RNAs (lncRNAs) participate in the regulation of a diverse range of biological processes. To explore the potential lncRNAs involved in the adaptation to high-altitude hypoxia of Tibetan sheep, we analysed the expression profile of lncRNAs and mRNAs in the liver and lung tissues of sheep using comparative transcriptome analysis between four Tibetan sheep populations (high altitude) and one Hu sheep population (low altitude). The results showed a total of 7848 differentially expressed (DE) lncRNA transcripts, and 22,971 DE mRNA transcripts were detected by pairwise comparison. The expression patterns of selected mRNAs and lncRNAs were validated by qRT-PCR, and the results correlated well with the transcriptome data. Moreover, the functional annotation analysis based on the Gene Ontology (GO) and Kyoto Encyclopedia of Genes and Genomes (KEGG) databases showed that DE mRNAs and the target genes of the lncRNAs were significantly enriched in organ morphogenesis, response to stimulus, haem binding, the immune system, arginine and proline metabolism, and fatty acid biosynthesis. The prediction of mRNA–mRNA and lncRNA–mRNA interaction networks further revealed transcripts potentially involved in adaptation to high-altitude hypoxia, and the hub genes *DDX24*, *PDCD11*, *EIF4A3*, *NDUFA11*, *SART1*, *PRPF8* and TCONS_00306477, TCONS_00306029, TCONS_00139593, TCONS_00293272, and TCONS_00313398 were selected. Additionally, a set of target genes, *PIK3R1*, *IGF1R*, *FZD6*, *IFNB2*, *ATF3*, *MB*, *CYP2B4*, *PSMD13*, and *TGFB1,* were also identified as candidate genes associated with high-altitude hypoxia adaptation. In conclusion, a collection of novel expressed lncRNAs, a set of target genes and biological pathways known to be relevant for altitude adaptation were identified by comparative transcriptome analysis between Tibetan sheep and Hu sheep. Our results are the first to identify the characterization and expression profile of lncRNAs between Tibetan sheep and Hu sheep and provide insights into the genetic regulation mechanisms by which Tibetan sheep adapt to high-altitude hypoxic environments.

## Introduction

Tibetan sheep are one of China's three primitive sheep populations and are mainly distributed among the Qinghai-Tibetan Plateau, with Qinghai, Tibet and Gannan in Gansu^1,2^. The combined effects of cold and hypoxia in high-altitude areas pose severe physiological challenges for Tibetan sheep. The decrease in oxygen partial pressure at high altitude reduces the oxygen supply to the cells in tissues of the respiratory system^3^. Decreased tissue oxygenation will severely limit aerobic metabolism and inhibit the ability to produce heat, thereby resisting the effects of the decrease in ambient temperature that accompanies an increase in altitude on the body^4,5^. Cold and hypoxic conditions at high altitudes have seriously affected the metabolic needs of Tibetan sheep^6^. Therefore, it is an interesting scientific question to analyse the adaptive evolution mechanism of Tibetan sheep in high cold and hypoxic environments. Elucidating the molecular mechanism by which Tibetan sheep adapt to extreme cold and hypoxia is a scientific goal of evolutionary genetics.

Oxygen is the key substance for an animal to carry out metabolism and maintain life, and it is the first need for life activities^7^. The inhaled oxygen is converted into available oxygen in the animal, and this is called blood oxygen. The blood oxygen carried by the blood provides energy to the whole body, and the amount of blood oxygen that is delivered is closely related to the working state of the heart and brain. The stronger the heart is able to pump blood, the higher the blood oxygen content. If the coronary blood transfusion ability of the heart is strong, then the concentration of blood oxygen that is delivered to the heart, brain and whole body is increased. Thus, the operating state of important animal organs is improved^8^. Medically, an area with an altitude of more than 3000 m is called a plateau. The impact of the plateau environment on animals involves many aspects, such as atmospheric geography, geochemistry, ecology, and other factors. Factors such as low atmospheric pressure, low oxygen, low temperature, low humidity, solar radiation, airflow, rainfall, light, wind, and snow often act on animals. Among them, the impact of plateau hypoxia has attracted wide attention from scholars because of its highly significant impact^3,9^.

In terms of molecular research on hypoxia adaptation, studies have shown that hypoxia inducible factor-1 (*HIF-1*) is one of the genes selected by the high altitude and low oxygen environment^10,11^. The *HIF-1α* subunit is encoded by the *HIF-1A* gene, which is primarily regulated by oxygen concentration and determines the level of *HIF-1α* activity^12^. The expression level of *HIF-2α* mRNA in Tibetan sheep was higher in lung, liver, kidney, and myocardial tissues than that in low-altitude sheep. It can quickly induce the expression of its downstream factors *VEGF* (vascular endothelial growth factor), *EPO* (erythropoietin), and lung surfactant and strengthen the lipid oxidative phosphorylation reaction. Thus, it can enhance the lung ventilation capacity, heart pumping function, kidney *EPO* synthesis and liver metabolic reaction to meet the oxygen and energy required for activity. The expression level of *STAT3* mRNA in Tibetan sheep is higher in lung, liver, and myocardial tissues than that in low-altitude sheep^13^. This indicates that the hypoxic environment increases the expression of *STAT3* protein to promote the generation of activated *STAT3*, which can effectively regulate the transcription of *VEGF*, *COX-2*, *MnSOD*, *UCP1*, *HIF-1α* and its downstream target genes. This results in enhanced ventilation capacity of Tibetan sheep lungs, hypoxic adaptability of the heart pumping function and energy metabolism^14^.

At present, several scientists have studied the mechanism of hypoxic adaptation in the Tibetan Plateau at the genetic level, but the molecular mechanism of Tibetan by which sheep adapt to this extreme environment, especially the role of genetic regulation, is still unknown. Long non-coding RNAs (lncRNAs) are transcripts longer than 200 nucleotides that can regulate mRNA expression at both the posttranscriptional and transcriptional levels^15^. There is limited literature on the detailed functional roles of lncRNAs in the high-altitude adaptation of Tibet^16–18^. The role of lncRNAs in hypoxic adaptation in Tibetan sheep has not yet been reported. The recent advancement in research of high-throughput sequence technology has introduced a new path to explore the molecular genetic basis of adaptive physiological traits. Transcriptome sequencing (mRNA and lncRNA) can not only identify important genes that cope with physiological challenges but also provide an in-depth understanding of the role of transcription regulation in adapting to evolutionary variation.

In this study, the genetic expression and regulation mechanisms of different populations and tissues in Tibetan sheep under different altitude conditions were investigated using comparative transcriptome analysis. Functional genes associated with adaptation to high-altitude hypoxia in Tibetan sheep were identified using mRNA, lncRNA and coexpression network analysis.

## Methods

### Ethics statement

All the experimental procedures mentioned in the present study were approved by the Science Research Department (in charge of animal welfare issues) of the Institute of Animal Sciences, Chinese Academy of Agricultural Sciences (IAS-CAAS). We obtained written informed consent to use the animals in this study from the owners of the animals, and all experiments on animals were conducted under a permit approved by the ethics committee of Lanzhou Institute of Husbandry and Pharmaceutical Sciences, Chinese Academy of Agricultural Sciences (Approval No. SYXK-2014-0002). All animals were handled in strict accordance with the recommendations in the Regulations for the Administration of Affairs Concerning Experimental Animals of the State Council of the People’s Republic of China. This study was carried out in compliance with the ARRIVE guidelines.

### Animal materials and sampling

We declare that we have no financial or personal relationships with other people or organizations that can inappropriately influence our work, and there is no professional or other personal interest of any nature or kind in any product, service and/or company that could be construed as influencing the position. Five sheep (*Ovis aries*) populations, including Huoba (HB, altitude 4468 m), Awang (AW, altitude 4452 m), Ganjia (GJ, altitude 3851 m) and Qilian (WT, altitude 3621 m) Tibetan sheep on the Qinghai-Tibet Plateau and Hu sheep (HS, altitude − 67 m), were obtained from individual farmers in this study. All the experimental sheep were raised under an environment with natural light and free access to food and water. Adult individuals (ram, aged 3–4 years) within each population were randomly selected for this study. A total of 60 sheep from 5 populations were used for physiological and biochemical characteristic analyses. All experimental animals were anaesthetized by pentobarbital sodium and then sacrificed after exsanguination. Three sheep within each population were selected to obtain liver and lung samples for transcriptome analysis (Table 1), and all the samples were immediately snap-frozen in liquid nitrogen for total RNA extraction.

**Table 1:** Sampling information of five sheep populations (*Ovis aries*) for transcriptome analysis.

Populations	Population code	Sample size	Sex	Longitude and latitude	Sampling location	Altitude (m)	Liver sample ID	Lung sample ID
Awang sheep	AW	3	Male	N:30°35′709″ E:098°27′755″	Ngawang third village, Awang Town, Gongjue County, Nyingchi prefecture of Tibet Autonomous Region	4452	L01	L16
							L02	L17
							L03	L18
Huoerba sheep	HB	3	Male	N:30°10′47″ E:083°10′09″	Zazi village, Horba township, Zhongba County, Shigatse prefecture of Tibet Autonomous Region	4468	L04	L19
							L05	L20
							L06	L21
Ganjia sheep	GJ	3	Male	N:35°21′017″ E:102°30′009″	Renqing village, Ganjia Town, Xiahe County, Gannan Tibetan Autonomous State, Gansu Province	3851	L10	L22
							L11	L23
							L12	L24
Qilian sheep	WT	3	Male	N:37°25′068″ E:099°00′905″	Awugeer village, Shengge Town, Qilian County, Delingha City, Mongolian Autonomous State, Qinghai Province	3620	L07	L25
							L08	L26
							L09	L27
Hu sheep	HS	3	Male	N:34°18′693″ E:117°57′763″	No. 169, Guzi village, Zhaodun town, Pizhou city, Jiangsu province	− 67	L13	L28
							L14	L29
							L15	L30

The number of altitude only refers to the value in sampling location, more animal samples from the same area were used for physiological and biochemical analysis.

### Physiological and biochemical measurement

To observe the changes in related blood physiological and biochemical parameters under hypoxia at high altitude in sheep, we sampled Tibetan sheep and Hu sheep from different altitudes. Jugular venous blood samples from 3- to 4-year-old adult sheep that lived at different altitudes, as described in Table 1, were collected to measure the haematological parameters. Red blood cell count (RBC), white blood cell count (WBC), haemoglobin content (HGB) and other parameters were measured using an HC-3000 Auto Hematology Analyzer (Jinan Meiyyilin Electronic Instrument Co., Ltd, Jinan, China China). Moreover, blood gas indices, including partial pressure of carbon dioxide (*P*CO_2_), oxygen partial pressure (*P*O_2_), oxygen saturation (O_2_S), and standard base excess (SBE), were repeatedly measured on an IL 1302 pH/blood gas analyser (Instrumentation Laboratories, MA). For the analysis of blood biochemical indices, the blood samples were centrifuged at 3000*g* for 10 min, after which the supernatant was collected. The serum biochemical parameters alanine aminotransferase (ALT), aspartate aminotransferase (AST), total protein (TP), plasma cholinesterase (PChE) and others were analysed with a Dirui CS-600 automatic biochemical analyser (Dirui Industrial Co., Ltd; Changchun, China).

After euthanasia, the lung organs were removed immediately and excised into 7 pieces of tissue from the same part of the left lung of each sheep. The lungs were washed with 0.9% saline and then fixed with Bouin’s solution containing 75 mL saturated picric acid solution, 25 mL formaldehyde, and 5 mL glacial acetic acid for 48 h at room temperature. Next, the tissues were embedded in paraffin, and 4-μm-thick sections were made for haematoxylin–eosin (HE) staining. The morphology of lung tissues was observed under a virtual microscope (Olympus, BX51, Japan)^19^.

### RNA isolation, library construction and sequencing

Total RNA was extracted using TRIzol Reagent (Life Technologies) according to the manufacturer’s protocol. RNA degradation and contamination, especially DNA contamination, were monitored on 1.5% agarose gels. RNA concentration and purity were measured using a NanoDrop 2000 Spectrophotometer (Thermo Fisher Scientific, Wilmington, DE). RNA integrity was assessed using the RNA Nano 6000 Assay Kit of the Agilent Bioanalyzer 2100 System (Agilent Technologies, CA, USA).

A total amount of 1.5 μg RNA per sample was used as input material for rRNA removal using the Ribo-Zero rRNA Removal Kit (Epicentre, Madison, WI, USA). Sequencing libraries were generated using the NEBNext Ultra Directional RNA Library Prep Kit for Illumina (NEB, USA) following the manufacturer’s recommendations, and index codes were added to attribute sequences to each sample. Briefly, fragmentation was carried out using divalent cations under elevated temperature in NEBNext First Strand Synthesis Reaction Buffer (5 ×). First strand cDNA was synthesized using random hexamer primers and reverse transcriptase. Second-strand cDNA synthesis was subsequently performed using DNA Polymerase I and RNase H. The remaining overhangs were converted into blunt ends via exonuclease/polymerase activities. After adenylation of the 3′ ends of DNA fragments, the NEBNext adaptor with a hairpin loop structure was ligated to prepare hybridization. To preferentially select insert fragments 150–200 bp in length, the library fragments were purified with AMPure XP Beads (Beckman Coulter, Beverly, USA). Then, 3 μL USER Enzyme (NEB, USA) was used with size-selected, adaptor-ligated cDNA at 37 °C for 15 min before PCR. Then, PCR was performed with Phusion High-Fidelity DNA polymerase, universal PCR primers and Index (X) Primer. Finally, PCR products were purified (AMPure XP system), and library quality was assessed on an Agilent Bioanalyzer 2100 and qPCR^20^. The libraries were sequenced on an Illumina HiSeq 2500 platform (Illumina Inc., San Diego, CA, USA) according to the manufacturer’s instructions, and 125 bp paired-end reads were generated.

### Quality control and RNA-seq data analysis

Raw data (raw reads) in fastq format were first processed through in-house Perl scripts. In this step, clean data (clean reads) were obtained by removing reads containing adapters, reads containing poly-N and low-quality reads from raw data. Additionally, the Q20, Q30, GC content and sequence duplication level of the clean data were calculated. All downstream analyses were based on clean data with high quality^21,22^. The clean reads were aligned to the genome using TopHat version 2.0.9. Mapped reads from TopHat for each sample were assembled using Cufflinks vision 2.1.1. The multiple assembled transcript files (GTF format) for different samples were then merged together to produce a unique transcriptome set using the Cuffmerge utility provided by the Cufflinks package.

### LncRNA analysis and quantification of gene expression levels

The transcriptome was assembled using Cufflinks and Scripture based on the reads mapped to the reference genome. The assembled transcripts were annotated using the Cuffcompare program from the Cufflinks package. The unknown transcripts were used to screen for putative lncRNAs. Four computational approaches, including CPC/CNCI/CPAT/Pfam, were combined to sort non-protein-coding RNA candidates from putative protein-coding RNAs in the unknown transcripts. Putative protein-coding RNAs were filtered out using a minimum length and exon number threshold. Transcripts with lengths greater than 200 nt and more than two exons were selected as lncRNA candidates and further screened using CPC/CNCI/CPAT/Pfam, which has the power to distinguish protein-coding genes from noncoding genes. In addition, different types of lncRNAs, including lincRNAs, intronic lncRNAs, and antisense lncRNAs, were selected using Cuffcompare.

Cuffdiff (v2.1.1) was used to calculate fragments per kilobase of exon per million fragments mapped (FPKMs) of both lncRNAs and coding genes in each sample^23^. Gene FPKMs were computed by summing the FPKMs of transcripts in each gene group based on the length of the fragments and read count mapped to this fragment.

### Differential expression analysis

Differential expression analysis of five groups was performed using the DESeq R package^24^. DESeq provides statistical methods for determining differential expression in digital gene expression data using a model based on the negative binomial distribution. The resulting *P*-values were adjusted using Benjamini and Hochberg’s approach for controlling the false discovery rate. Genes with an adjusted *P*-value < 0.01 and absolute value of log2 (fold change) > 1 found by DESeq were assigned as differentially expressed.

### Prediction of potential target genes of lncRNAs

To explore the roles of lncRNAs in Tibetan sheep adaptation to hypoxia at high altitude, lncRNA-targeted genes were predicted based on the cis and trans principles. Cis action indicated a positional relationship, representing a gene located within the range of 100 kb from the lncRNA. Trans action meant that there were complementary sequences between the mRNA and lncRNA; the sequences of mRNAs that overlapped with lncRNAs were predicted by LncTar software^25^. These two interaction mechanisms were considered preferential for the prediction of lncRNA-targeted genes.

### Functional enrichment analysis

Gene functional enrichment analysis included Nr (NCBI nonredundant protein sequences), COG (clusters of orthologous groups of proteins), Swiss-Prot (a manually annotated and reviewed protein sequence database), GO (Gene Ontology; http://www.geneontology.org/), and KEGG^26^ (Kyoto Encyclopedia of Genes and Genomes; http://www.genome.jp/kegg/) analyses^27^, which were utilized for screening genes related to highland hypoxia. GO enrichment analysis of the differentially expressed genes was implemented by the topGO R packages^28^. KOBAS software^29^ was used to test the statistical enrichment of differentially expressed genes (DEGs) in KEGG pathways.

### Interaction network construction

Protein–protein interaction analysis of DEGs was based on the commonly used STRING database^30^. Briefly, the sequences of differentially expressed lncRNA targets were blasted (blastx) to the *Ovis aries* genome to predict protein–protein interactions using the STRING database. Then, the mRNA–mRNA and lncRNA–mRNA interaction networks were visualized by using Cytoscape version 3.5.1^31^.

### Quantitative real-time PCR (qRT-PCR) validation

Several DE lncRNAs, target genes and mRNAs putatively associated with hypoxia responses at high altitudes were selected and confirmed by qRT-PCR with GAPDH used as an internal reference. The primers used for qRT-PCR are listed in Table S1. qRT-PCR was carried out using a Roche LightCycler 96 using iTaq Universal SYBR Green Supermix (Bio-Rad, United States). Each real-time RT-PCR (in 25 μL) involved 12.5 μL 2 × SYBR Green Real-time PCR Master Mix (TaKaRa, Dalian), 1 μL of each primer, 2 μL cDNA and 8.5 μL H_2_O. The amplification procedures were 95 °C for 5 min initially, followed by 45 cycles of 95 °C for 15 s and 60 °C for 1 min. Quantification of mRNAs and lncRNAs was performed using the standard curve method with average cycle thresholds (Ct). The qRT-PCR data were generated from three independent samples per group. The correlation between the results of RNA-seq and qPCR was calculated using a correlation test.

### Statistical analysis

All data was presented as the mean ± SD. The Statistical Program for Social Sciences (SPSS) 20.0 software was employed to perform all of the statistical analyses. The multigroup comparisons of the means were analysed using one-way analysis of variance. Statistically significant differences were determined at a *p*-value < 0.05.

## Results

### Physiological and biochemical indices of five sheep populations from regions at different altitudes

**Figure 1:**
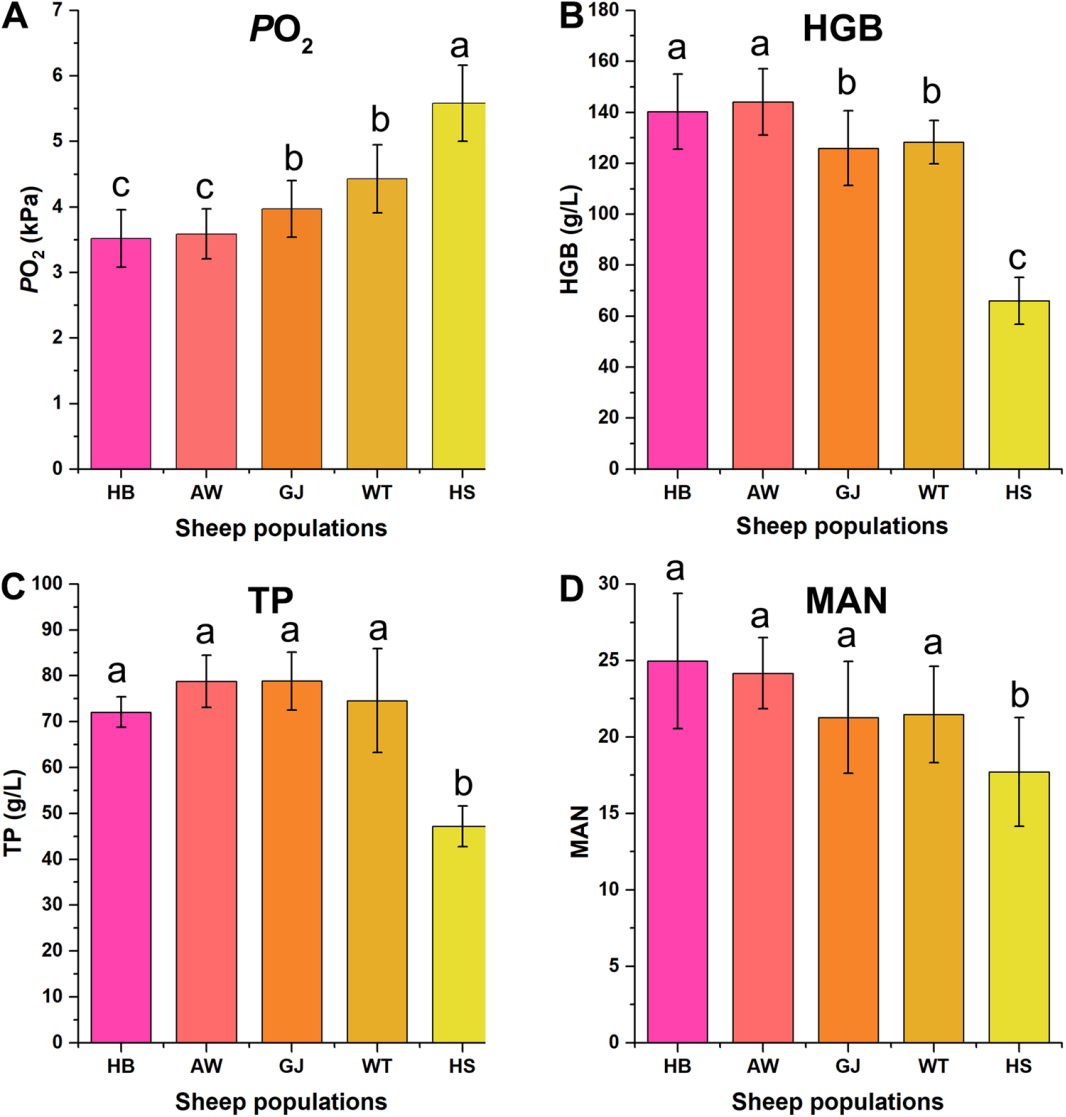
The physiological and biochemical indices of five sheep populations from different altitude. (**A**) Partial pressure of carbon dioxide (*P*O_2_). (**B**) Haemoglobin contents (HGB). (**C**) Total protein (TP). (**D**) The number of alveolar counted per unit area (MAN). The columns with different letters are significantly different at *P* < 0.05.

Physiological and biochemical indices were measured and are shown in Table S2 and Fig. 1. The results show that the lungs of Tibetan sheep from regions of high altitude have typical tissue structure characteristics. Compared with Hu sheep from low-altitude regions, the terminal bronchioles of Tibetan sheep had a larger diameter, an increased thickness of the alveolar septum, and an increased number of alveoli and blood vessels per unit area (*P* < 0.05). Furthermore, the measurement results from the blood gas indices of Tibetan sheep at different altitudes found that the *P*O_2_, O_2_S, and SBE decreased significantly as the altitude increased (*P* < 0.05).

In addition, the results showed that the HGB, RBC, white WBC, and other indices of Tibetan sheep at high altitude were significantly higher than those of low-altitude Hu sheep (*P *< 0.05), and the red blood cell distribution width coefficient of variation (RDW-CV) decreased. The measurement results from serum biochemical indices showed that AST, TP, and others increased with altitude (*P *< 0.05), while ALT and PChE decreased.

### Identification of lncRNAs and mRNAs in sheep liver and lung tissue

The Illumina sequencing of cDNA libraries from 30 samples derived from five sheep populations yielded a total of 187.28 Gb and 190.63 Gb clean data for liver and lung tissue, respectively. At least 78.05% of the clean reads from each sample were mapped to the sheep reference genome (Oar_v3.1, ftp://ftp.ensembl.org/pub/release-78/fasta/ovis_aries/), of which more than 78.52% were uniquely mapped (Table S3).

**Figure 2:**
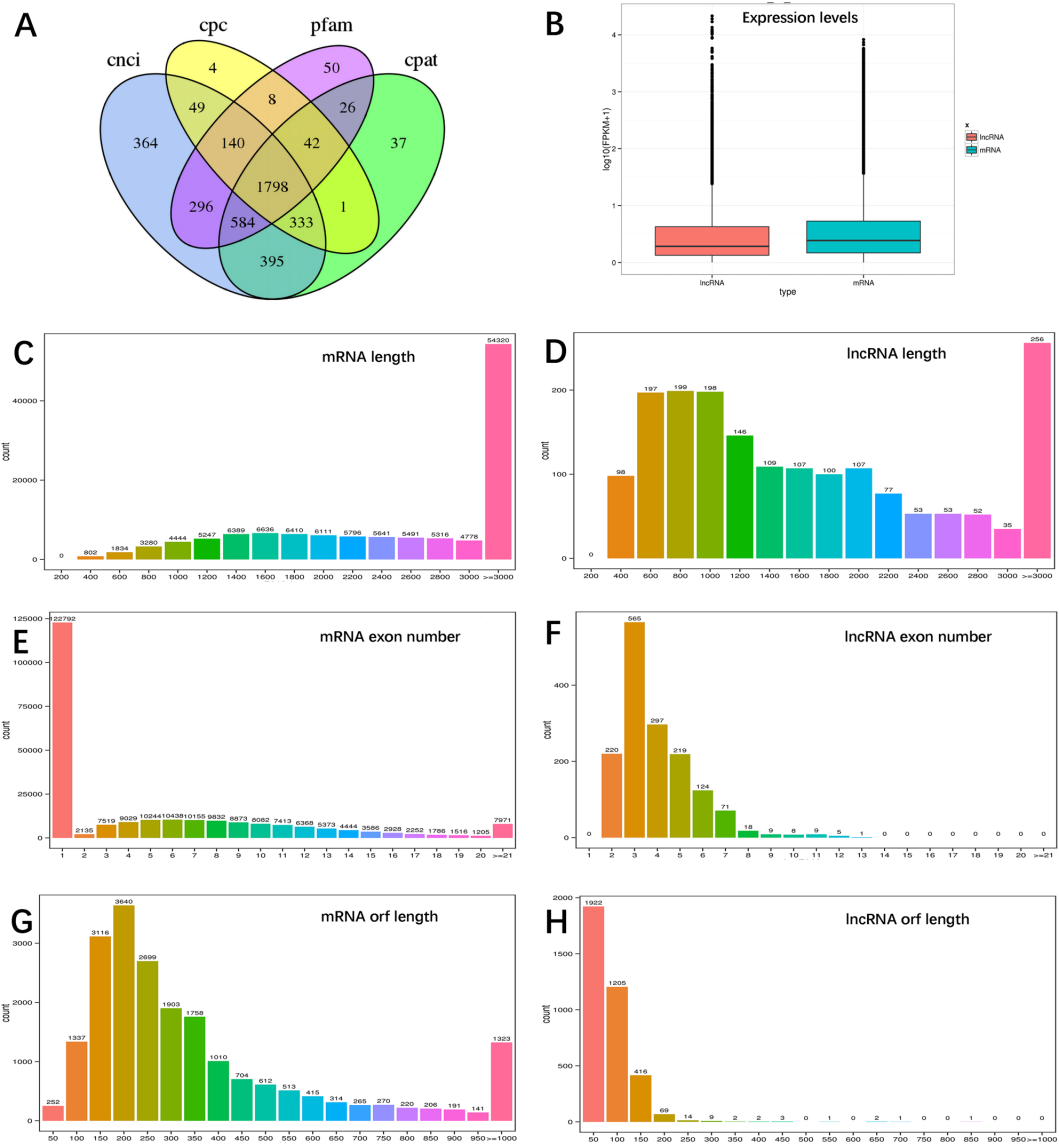
Identification of lncRNAs and mRNAs involved in high altitude hypoxia adaptation in lung tissue. Coding potentiality filter using CPAT/CNCI/CPC/Pfam (**A**). The boxplot shows the expression levels (log10 (FPKM + 1)) of lncRNAs and mRNAs (**B**). Transcript lengths distribution of mRNAs (**C**) and lncRNAs (**D**). Exon number distribution of mRNAs (**E**) and lncRNAs (**F**). Open reading frame (ORF) length distribution of mRNAs (**G**) and lncRNAs (**H**).

In the liver samples, a total of 6249 lncRNAs were identified after coding potential filters using CNCI, CPC, CPAT and Pfam-scan software, and 20,078 mRNAs and 2728 novel transcripts were identified. In addition, a total of 1798 lncRNAs, 13,319 mRNAs and 750 novel transcripts were identified from the lung samples (Table S4). The majority of lncRNAs comprised two or three exons, whereas mRNAs contained a broad range of exon numbers from 2 to 30. The transcript length and ORF length of lncRNAs were significantly shorter than those of mRNAs. Moreover, the expression levels of mRNAs and lncRNAs were further analysed using FPKM. The mRNA transcript levels were higher than those of lncRNAs in both liver (Fig. S1) and lung (Fig. 2) tissue.

### DE lncRNA and mRNA between Hu sheep and Tibetan sheep

The correlation coefficient between samples of the same group was trending towards 1; thus, it is reasonable to perform further data analyses (Fig. S2). Based on a fold change of ≥ 2 and false discovery rate of < 0.05, in total, 7848 differentially expressed (DE) lncRNA transcripts and 22,971 DE mRNA transcripts were detected in all groups by pairwise comparison (Table S5).

**Figure 3:**
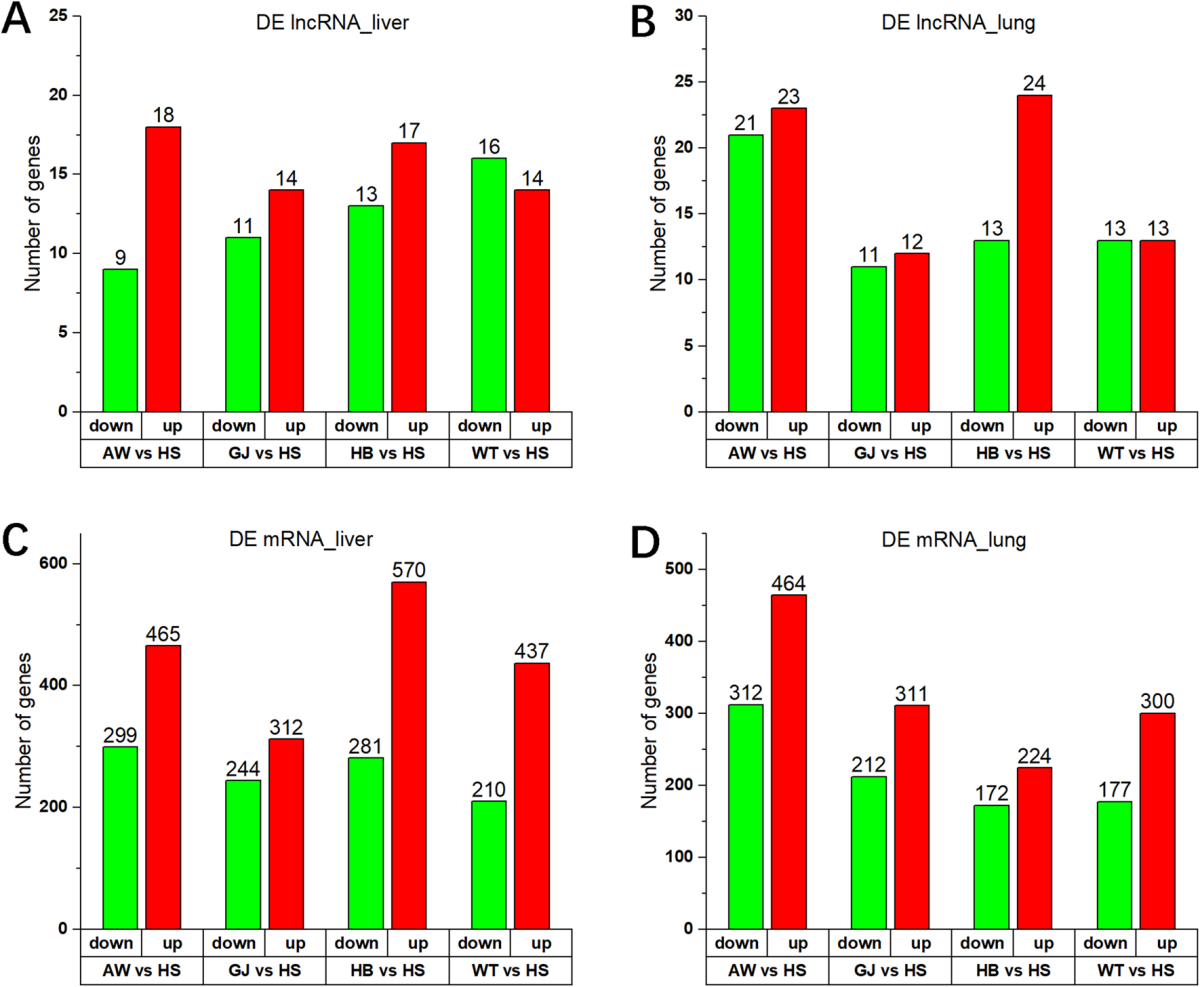
The up and down regulation of DE genes of five sheep populations. (**A**) Number of DE lncRNAs in liver samples. (**B**) Number of DE lncRNAs in lung samples. (**C**) Number of DE mRNAs in liver samples. (**D**) Number of DE mRNAs in lung samples. Red: up-regulated; Green: down-regulated.

**Figure 4:**
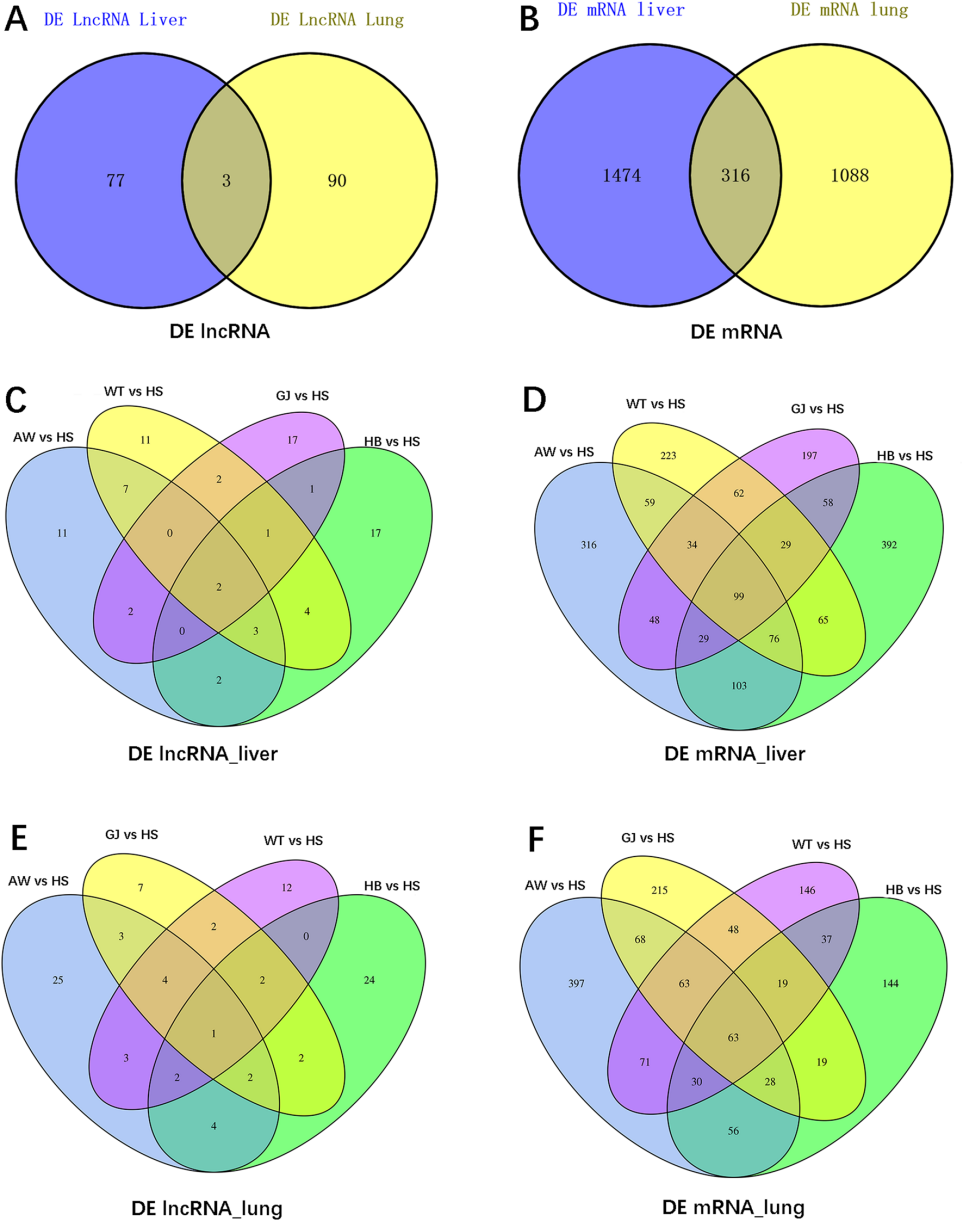
Venn diagram of common differential expression genes. (**A**) The common DE lncRNAs in pairwise comparison between lung and liver tissue. (**B**) The common DE mRNAs in pairwise comparison between lung and liver tissue. (**C**) The common DE lncRNAs in four comparison groups of liver samples (AW vs. HS, HB vs. HS, GJ vs. HS, WT vs. HS). (**D**) The common DE mRNAs in four comparison groups of liver samples (AW vs. HS, HB vs. HS, GJ vs. HS, WT vs. HS). (**E**) The common DE lncRNAs in four comparison groups of lung samples (AW vs. HS, HB vs. HS, GJ vs. HS, WT vs. HS). (**F**) The common DE mRNAs in four comparison groups of lung samples (AW vs. HS, HB vs. HS, GJ vs. HS, WT vs. HS).

Furthermore, we focused on pairwise comparisons between Tibetan sheep and Hu sheep (AW vs. HS, HB vs. HS, GJ vs. HS, WT vs. HS). The up- and downregulation of lncRNAs and mRNAs are shown in Fig. 3 and Table S6, and their distribution on chromosomes is shown in Fig. S3. The analysis of common DE genes between the liver and lung by pairwise comparison found 3 common DE lncRNAs and 316 common DE mRNAs (Fig. 4A,B). Further analysis of common differentially expressed genes (DEGs) among Tibetan sheep and Hu sheep detected 2 common DE lncRNAs and 99 common DE mRNAs in the liver by pairwise comparison (Fig. 4C,D). Additionally, 1 common DE lncRNA transcript and 63 common DE mRNA transcripts were detected in the lung (Fig. 4E,F).

### Functional annotation of DE mRNAs and the target genes of DE lncRNAs

The overall functional annotation of DE mRNAs in pairwise comparisons (AW vs. HS, HB vs. HS, GJ vs. HS, WT vs. HS) is described in Table S7. As lncRNAs could exert effects on cis-acting or trans-acting target genes, we predicted 1739 target genes of DE lncRNAs based on the cis and trans RNA–RNA interaction principle. In total, 218 and 197 target genes were differentially expressed and annotated from liver and lung tissue by pairwise comparison (AW vs. HS, HB vs. HS, GJ vs. HS, WT vs. HS) (Table S7).

To elucidate the functions of DE mRNAs, GO enrichment analysis was performed using topGO to search for the most significant GO terms of DE mRNAs. The most enriched GO terms of the DE mRNAs in each comparison are shown in Table S8. All of the DE mRNAs were assigned to GO terms of biological processes, cellular components, and molecular function. Moreover, response to stimulus, tissue development, skeletal system development, response to stress, regulation of signal transduction, organ development, antioxidant activity, calcium ion binding, haem binding, and lipid antigen binding were identified as significantly enriched GO terms. According to the KEGG analysis, the most enriched pathway to DE mRNAs (*P* < 0.05) was predominantly associated with amino acid metabolism, lipid metabolism and the immune system, such as arginine and proline metabolism, haematopoietic cell lineage, fatty acid biosynthesis, glutathione metabolism, and biosynthesis of unsaturated fatty acids.

### LncRNA–mRNA co-expression networks construction

To understand the effects of lncRNAs on the regulation of target genes, we constructed an mRNA–lncRNA regulatory network. First, we selected the target genes of DE lncRNAs to construct functional networks by referring to the STRING database, with each gene corresponding to a node. Two genes were connected by an edge, indicating a correlation between mRNA–mRNA. Next, DE lncRNAs and their corresponding target genes were used to construct the lncRNA–mRNA interaction network. Then, the mRNA–mRNA and lncRNA–mRNA networks were merged (Fig. S4). Within the network analysis, we focused on the top 10 DE mRNAs with the highest degree and lncRNAs that interacted with more target genes (Table S9), which probably constitute the core of the network. The results implied that the DE mRNAs *DDX24*, *PDCD11*, *EIF4A3*, *NDUFA11*, *SART1*, and *PRPF8* and the DE lncRNAs TCONS_00306477, TCONS_00306029, TCONS_00139593, TCONS_00293272, and TCONS_00313398 might play key roles in adaptation to hypoxia. Although these lncRNAs require further experimental validation, this information might be helpful to explore the potential mechanisms involved in the adaptation to hypoxia at high altitudes.

### Candidate genes screened for adaptation to hypoxia at high altitudes

A set of candidate genes that were putatively associated with hypoxia responses to high altitudes were screened from the target genes of DE lncRNAs. For a detailed description of the relevant functions and pathways of the candidate genes, see Table 2. Among them, three (*PIK3R1*, *IGF1R* and *PDK1*) were in the classical HIF-1 pathway; five were found in the corresponding downstream vascular endothelial growth factor (*FZD4* and *IFNB2*) and glycolysis/gluconeogenesis pathways (*ATF3*, *PPCK1* and *PFKFB2*). The dysregulation of genes in these relevant pathways indicates that hypoxia-induced factors, angiogenesis, and glycolysis metabolism are the most important factors that allow sheep to manage extreme hypoxic environmental pressure. We also found six genes (*MB*, *CYP2C31*, *CYP2B4*, *CYP2B5*, *CYP1A1*, and *CYCS*) that were functionally involved in oxygen binding, oxygen transport, and haem binding.

**Table 2:** The candidate genes putatively associated with the plateau adaptation of Tibetan sheep.

Gene ID	Gene symbol	Gene description
ENSOARG00000006910	MB	Oxygen binding
ENSOARG00000005870	PIK3R1	HIF-1 signaling pathway
ENSOARG00000007525	IGF1R	HIF-1 signaling pathway
ENSOARG00000018633	PDK1	HIF-1 signaling pathway
ENSOARG00000000709	CYP2C31	Heme binding
ENSOARG00000007211	CYP2B4	Heme binding
ENSOARG00000007575	CYP2B5	Heme binding
ENSOARG00000007316	CYP2B11	Heme binding
ENSOARG00000012689	CYP1A1	Heme binding
ENSOARG00000025165	CYCS	Heme binding
ENSOARG00000004175	FZD6	Vascular endothelial growth factor
ENSOARG00000008791	IFNB2	Vascular endothelial growth factor
ENSOARG00000010493	ATF3	Glycolysis/gluconeogenesis
ENSOARG00000017498	PPCK1	Glycolysis/gluconeogenesis
ENSOARG00000007023	PFKFB2	Glycolysis/gluconeogenesis
ENSOARG00000001159	MMP14	Response to stress
ENSOARG00000007468	TGFB1	Immune function
ENSOARG00000002665	TUBB4B	Cytoskeleton
ENSOARG00000008674	PSMD13	Proteasome regulatory particle
ENSOARG00000002857	COL3A1	Collagen involved pathways
ENSOARG00000007594	COL1A2	Collagen involved pathways
ENSOARG00000006399	DSG3	Keratinocyte adhesion in skin
ENSOARG00000008745	ATP6	Mitochondrial ATPase assembly

Moreover, we found that the GO terms of the target genes were primarily associated with metabolic processes (amino acids, lipids, and fatty acids), regulation of immune system processes, immune response, biological regulation, response to stress and response to stimulus (Table S7). These functional terms are biologically relevant to plateau adaptations because they are involved in energy metabolism, immune function, oxidation reaction, and stress response, which are important regulating factors of the response of Tibetan sheep to extreme hypoxic environments.

### Validation of DE mRNAs and lncRNAs by qRT-PCR

**Figure 5:**
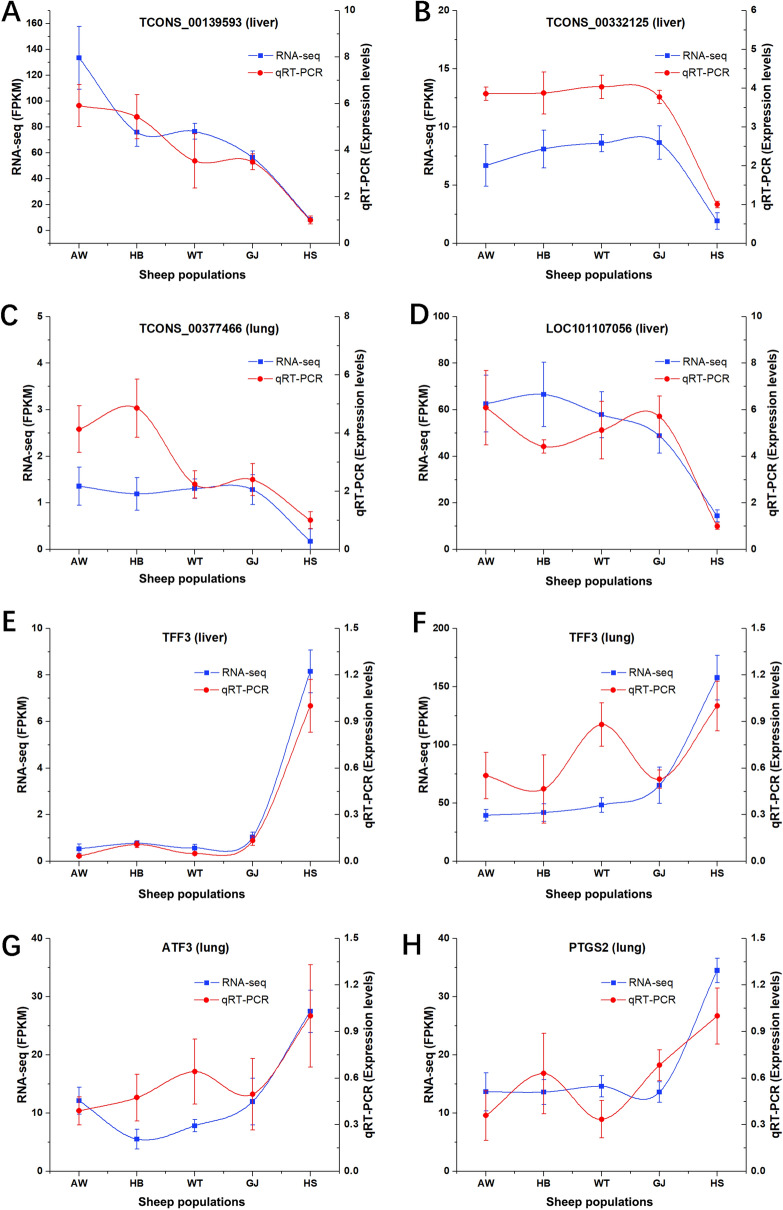
Confirmation of expression patterns of the eight selected differential expression genes using qRT-PCR. The trends of qRT-PCR results are consistent with RNA-seq data. (**A**) The common DE lncRNA TCONS_00139593 in five sheep populations of liver samples (AW, HB, GJ, WT, HS). (**B**) The common DE lncRNA TCONS_00332125 in five sheep populations of liver samples. (**C**) The common DE lncRNA TCONS_00377466 in five sheep populations of lung samples. (**D**) The common DE lncRNA TCONS_101107056 in five sheep populations of liver samples. (**E**) The common DE mRNAs TFF3 in five sheep populations of liver samples. (**F**) The common DE mRNAs TFF3 in five sheep populations of lung samples. (**G**) The common DE mRNAs ATF3 in five sheep populations of lung samples. (**H**) The common DE mRNAs PTGS2 in five sheep populations of lung samples.

To evaluate the reliability of RNA sequencing, 7 DE lncRNAs, 13 target genes, and 20 DE mRNAs were selected and validated by qRT-PCR from different sheep groups. All levels of DE lncRNAs and mRNAs were consistent with the RNA-seq results, indicating that the RNA-seq data were reliable (Table S10). The qRT-PCR and RNA-seq results for the common DE lncRNAs TCONS_00139593 and TCONS_00332125 in the liver and TCONS_00377466 in the lung are shown in Fig. 5. Additionally, the confirmed results of 3 DE mRNAs that were enriched in the haem binding term (*PTGS2* and *LOC101107056*) and HIF-1 pathway (*TFF3*) are also shown in Fig. 5.

## Discussion

### Comparison of physiological and biochemical indices

At present, research on the adaptability of Tibetan sheep to hypoxia at high altitude has made important progress in histology, morphology, physiology, and anatomy^32–35^. Studies suggest that compared to Tan sheep living in low-altitude areas, Tibetan sheep have developed a cerebral arterial system in which the main arteries are thicker in diameter, and the collateral branches in the cerebral arteries are developed and stretched longer. There are many small arteries, and this feature is conducive to effective blood supply to the brain and the regulation of cerebral arterial blood pressure^36^. This might be one of the anatomical characteristics of Tibetan sheep that aids in adaptation to a high-altitude hypoxic environment. Anatomical studies on the vascular system of other tissues and organs of Tibetan sheep have similar results. For example, compared to small-tailed Han sheep, Tibetan sheep have more capillaries in the alveolar septum, and they are mostly open, which also increases with altitude. The alveolar septum is thick, indicating that the alveolar septum is rich in capillaries and elastic fibres. These structural features are conducive to increasing alveolar ventilation, increasing pulmonary blood flow, accelerating blood oxygen transport, and improving the lung gas exchange rate in a hypoxic environment. Compared to low-altitude sheep, Tibetan sheep have more red blood cells and higher haematocrit and haemoglobin contents. Under a low oxygen environment, Tibetan sheep mainly adapt to a low oxygen environment by increasing the haemoglobin content of the blood^37^.

In this study, we first examined the haematological changes and serum biochemical parameters in four Tibetan sheep and one Hu sheep population. In agreement with previous reports, the haematological parameters, serum biochemical parameters, blood gas indices and morphology of lung tissues showed significant changes between Tibetan sheep (high altitude) and Hu sheep (low altitude). The haematological parameters, including RBC, WBC, HGB, HCT, MCV, and PLT, became significantly higher as the altitude increased (*P* < 0.05). The reason for the difference might be due to the extreme cold and hypoxic factors, especially the value of HGB, which increases with increasing altitude. Under low *P*O_2_, HGB dissociates from oxygen to provide the body with the oxygen needed for energy metabolism to better adapt to the low oxygen environment. Biochemical parameters, including AST, TP, ALB, GLO, ALP, and LDH, significantly increased with increasing altitude, while ALT and PCHE decreased with increasing altitude. In particular, the values of TP increased with altitude, which is helpful to enhance the immune function of sheep and adapt to the high-altitude ecological environment. Moreover, the blood gas indices, including *P*CO_2_, *P*O_2_, O_2_S, SBC, TCO_2_, and SBE, all significantly decreased with increasing altitude. Related studies have shown that Tibetan sheep can reduce tissue oxygen demand and cell metabolism levels through specific physiological changes and adapt to the plateau hypoxic environment at the molecular level by regulating hypoxia inducible factor^13^. Additionally, the morphology of lung tissue was observed, and we found that the terminal bronchioles, the number of alveoli counted per unit area, the alveolar septum thickness and the number of vessels per unit area significantly increased with increasing altitude. These changes in tissue structure are conducive to accelerating blood oxygen transport and increasing alveolar ventilation. This increases the lung blood flow and lung gas exchange rate of Tibetan sheep in a hypoxic environment to a certain extent. Early studies have shown that these changes are the key characteristics of adaptation to high-altitude environments by Tibetan sheep^38,39^.

### Analysis of lncRNAs and their target genes

Due to the key roles of lncRNAs in many important biological processes, these are currently of particular interest^40,41^. The rapid development of high-throughput sequencing methods has led to the discovery of thousands of lncRNAs in recent years. Studies have reported that lncRNAs are involved in primary wool follicle induction in carpet wool sheep^42^, sheep fat-tail development^43^, sheep skeletal muscle development^44^, prolificacy in Hu sheep^45^ and sheep testicular maturation^19,42^ with high-throughput sequencing technology. However, the expression and function of lncRNAs in Tibetan sheep adaptation to high-altitude hypoxia are still unclear. To provide some insights into the biological functions of lncRNAs in Tibetan sheep adaption to high-altitude hypoxia, a comprehensive analysis of lncRNA and mRNA profiling data from Tibetan sheep and Hu sheep, together with data from a public database, was performed. We identified the core lncRNAs and their target genes and validated their expression by qRT-PCR. Overall, our work uncovered an interlaced transcript network that is involved in a high-altitude hypoxic environment.

The analysis of common DE genes among Tibetan sheep and Hu sheep found 2 common DE lncRNAs TCONS_00139593 and TCONS_00332125 in the liver and 1 common DE lncRNA TCONS_00377466 in the lung. The target genes of TCONS_00139593, including *DOCK11*, *CYP2B4*, *TACO1*, *CYP2B11*, *ATP5SL*, *B3GNT8 BCKDHA*, and *EXOSC4*, TCONS_00332125, including *TTR* and *DSG3*, TCONS_00377466, including *WDR77*, *ZMYND19*, and *MRPL41*, were found. Among them, *DSG3*, *CYP2B4* and *CYP2B11* are candidate genes related to high-altitude hypoxia adaptation, which are listed in Table 2. Moreover, the lncRNA–mRNA interaction network of liver samples showed that TCONS_00306477, TCONS_00306029, TCONS_00029720, TCONS_00145870, TCONS_00139593, TCONS_00380986, TCONS_00309307, TCONS_00225957, TCONS_00321529, and TCONS_00100469 interacted with more target genes and suggested hub genes related to high-altitude hypoxia adaptation. The lncRNA–mRNA interaction network in lung samples showed that TCONS_00293272, TCONS_00313398, TCONS_00344932, TCONS_00078812, TCONS_00352306, TCONS_00380999, TCONS_00088235, TCONS_00467816, TCONS_00078180, and TCONS_00315164 interacted with more target genes and suggested hub genes.

Preliminary research of candidate genes that are associated with hypoxia responses at high altitudes reported genome-wide scans that revealed positive selection in several regions that contained genes whose products are likely to be involved in high altitude adaptation^46^. Finally, a set of 247 functional candidate genes was identified. The functional candidate gene categories included detection of oxygen (GO: 0003032), NO metabolic process (GO: 0046209), oxygen sensor activity (GO: 0019826), oxygen binding (GO: 0019825), oxygen transport (GO: 0015671), oxygen transporter activity (GO: 0005344), response to hypoxia (GO: 0001666), response to oxygen levels (GO: 0070482), vasodilation (GO: 0042311), and hypoxia response via *HIF* activation (P00030) in the panther pathway. In this study, we found that target genes, including *MB*, *PIK3R1*, *CYP1A1*, *MMP14*, and *TGFB1,* belong to the list of 247 hypoxia genes. In addition, *MMP14*^47^, *TUBB4B*^48^, *PSMD13*^49^, *COL3A1*, *COL1A2*^50^, *DSG3*^51,52^, and *ATP6*^53^ were also identified as candidate genes associated with high-altitude adaptation by previous functional studies.

Myoglobin, encoded by *MB*, is a haemoprotein present in cardiac, skeletal and smooth muscle that serves as a reserve supply of oxygen and facilitates the movement of molecular oxygen from the cell membrane to mitochondria^54^. A previous study demonstrated that *PIK3R1*, which is involved in the *HIF-1α* signalling pathway, plays a critical role in mediating adipose tissue insulin sensitivity^55^. Another previous study showed that *CYP1A1* transcriptional activation was significantly decreased upon *PCB 126* stimulation under conditions of hypoxia. Additionally, hypoxia pretreatment reduced *PCB 126*-induced AhR binding to *CYP1* target gene promoters^56^. Additional research has shown that *MMP14* is upregulated under hypoxic conditions and that this occurs by the interaction of *HIF-1α* and the *MMP14* gene promoter region^57^. Chen et al*.* suggested that *TGF-β1* encoded by *TGFB1* decreases hypoxia–reoxygenation injury and attenuates alterations in NOS and PKB phosphorylation in myocytes exposed to hypoxia–reoxygenation^58^.

Yang et al*.*^59^ generated whole-genome sequences from 77 native sheep and detected a novel set of candidate genes as well as pathways and GO categories that were putatively associated with hypoxia responses at high altitudes. Specifically, several positively selected genes within or regulating the *HIF-1* pathway, the *VEGF* pathway, the *VSMC* pathway, glycolysis and lipids were identified for energy metabolism. The network of relevant pathways indicated that hypoxia-induced factors, angiogenesis, vasodilatation and glycolysis metabolism were the most important factors that allowed sheep to manage extreme hypoxic environmental pressure. Seven sheep breeds representing both highland and lowland breeds from different areas of China were genotyped for a genome-wide collection of single-nucleotide polymorphisms (SNPs)^60^. The detected SNPs were found in genes involved in angiogenesis, energy production and erythropoiesis and played a crucial role in hypoxia adaptation. Here, we found the target genes *PIK3R1*, *IGF1R* and *PDK1* in the classical *HIF-1* pathway and *FZD4*, *IFNB2, ATF3*, *PPCK1*, *PFKFB2* in the corresponding downstream *VEGF* and glycolysis/gluconeogenesis pathways, which played a central role in regulating cellular responses to hypoxia^46,61,62^. Hypoxia regulates *IGF1* expression through *HIF-1α*, and the inhibition of *HIF-1α* or *IGF1R* decreased CD133- and Oct4-positive GRPs under hypoxia^63^. Mora et al*.* found that the *PDK1* signalling network plays an important role in regulating cardiac viability and preventing heart failure, and the deficiency of *PDK1* in cardiac muscle results in heart failure and increased sensitivity to hypoxia^64^. *ATF3* is a stress-induced transcription factor that plays important roles in regulating immune and metabolic homeostasis. Overwhelming evidence confirms that the *ATF3* gene is activated in many tissues by a variety of stress signals, including proinflammatory cytokines, ischaemia and hypoxia^65^. Parra et al*.* found that the mRNA levels of the glycolytic markers *HK2*, *PFKFB2* and *GLUT1* increased in accordance with a metabolic shift towards nonmitochondrial ATP generation during hypoxia^66^. The *VEGF* pathway downstream of *HIF-1* and glycolysis is an important mechanism of energy metabolism in sheep under extreme hypoxic conditions. The dysregulation of genes in these pathways indicated that hypoxia-induced factors, angiogenesis, and glycolysis metabolism were the most important factors that allowed sheep to manage extreme hypoxic environmental pressure.

The *CYP2C31*, *CYP2B4*, *CYP2B5*, and *CYCS* genes were functionally involved in oxygen binding, oxygen transport, and haem binding. In humans, indirect evidence suggests that hypoxia reduces the rate of biotransformation of drugs cleared by the cytochrome P450 subfamilies *CYP1A*, *2B*, and *2C*. Fradette et al*.* found that hypoxia downregulates rabbit hepatic *CYP1A1*, *1A2*, *2B4*, *2C5*, and *2C16* and upregulates *CYP3A6*. *CYP3A11* and P-glycoprotein were upregulated in the livers of hypoxic rats^67^. In addition, *TUBB4B*, *PSMD13*, *COL3A1*, *COL1A2*, *DSG3,* and *ATP6* were also identified as candidate genes associated with high-altitude adaptation by previous functional studies. Kharrati-Koopaee et al*.* found that the *PSMD13* gene was associated with hypoxia by whole genome sequencing of lowland and highland chickens^49^. Qi et al*.* conducted a cross-tissue, cross-altitude, and cross-species study to characterize the transcriptomic landscape of domestic yaks. They found that the lung and heart are two key organs showing adaptive transcriptional changes, and five collagen genes (*COL1A2*, *COL3A1*, *COL5A2*, *COL14A1*, and *COL15A1*) highlight the crucial role of collagen-involved pathways in high-altitude adaptation^50^. Previous exome sequencing of five Chinese cashmere goat breeds revealed a candidate gene, *DSG3*, responsible for the high-altitude adaptation of the Tibetan goat. The mutations significantly segregated high- and low-altitude goats in two clusters, indicating the contribution of *DSG3* to high-altitude hypoxia adaptation in Tibetan goats^52^. Wang et al*.* sequenced the *ATP8* and *ATP6* genes in 66 Tibetan yaks and 81 domestic cattle and found that haplotypes H4 in *ATP8* and H5 in *ATP6* present only in Tibetan yaks were suggested to be positively associated with high-altitude adaptation^53^.

Overall, the expression profile of lncRNAs and mRNAs in liver and lung tissue determined by the comparative transcriptome analysis between high- and low-altitude sheep indicates that the lung and liver are two key organs that show adaptive transcriptional changes. Moreover, the candidate genes involved in HIF-1, VEGF, and glycolysis/gluconeogenesis pathways, as well as oxygen binding, oxygen transport, and haem binding molecular function that were putatively associated with hypoxia responses at high altitudes, were screened. These findings, in combination with the results of physiological and biochemical index analyses, are valuable for understanding the genetic mechanism of hypoxic adaptation in sheep. Nevertheless, a limitation for this study is the earlier versions of the sheep reference genome was used.

## Conclusions

In summary, we demonstrated the expression profiles of mRNAs and lncRNAs in Tibetan sheep and Hu sheep to understand their regulatory roles in adaptation to a high-altitude hypoxic environment by Tibetan sheep. A collection of novel expressed lncRNAs, a set of target genes and biological pathways known to be relevant for altitude adaptation were identified by comparative transcriptome analysis between Tibetan sheep and Hu sheep. Our study might contribute to determining the core DE lncRNAs between Tibetan sheep and Hu sheep. In addition, further study of these lncRNAs could provide useful insights into the genetic regulation mechanism of lncRNAs in adaption to a high-altitude hypoxic environment in Tibetan sheep.

## Supplementary Information

Supplementary Legends.

Supplementary Figure S1.

Supplementary Figure S2.

Supplementary Figure S3.

Supplementary Figure S4.

Supplementary Table S1.

Supplementary Table S2.

Supplementary Table S3.

Supplementary Table S4.

Supplementary Table S5.

Supplementary Table S6.

Supplementary Table S7.

Supplementary Table S8.

Supplementary Table S9.

Supplementary Table S10.

## Data Availability

The datasets generated and/or analysed during the current study are included in this published article and its supplementary information files. Genomic resources Oar_v3.1 for *Ovis aries* are available from Ensembl (ftp://ftp.ensembl.org/pub/release-78/fasta/ovis_aries/). Final sequences obtained from all samples were submitted to the SRA database of NCBI with accession number PRJNA791158 and PRJNA788998.

## Abbreviations

LncRNA: Long non-coding RNA
DEG: Differentially expressed gene
GO: Gene Ontology
KEGG: Kyoto Encyclopedia of Genes and Genomes
FPKM: Fragments per kilobase of exon per million fragments mapped
qRT-PCR: Quantitative Real-Time PCR
VEGF: Vascular endothelial growth factor
HIF-1: Hypoxia inducible factor-1
