# Supplementary material for: Comparative analysis of long noncoding RNA and mRNA expression provides insights into adaptation to hypoxia in Tibetan sheep
Source: Sci Rep. 2022 Apr 21;12:6597. doi: 10.1038/s41598-022-08625-y (PMC9023463; doi:10.1038/s41598-022-08625-y)
Supplement: Supplementary file 1 — Supplementary Legends. [file 41598_2022_8625_MOESM1_ESM.docx]

Figure and Table Legends of Supplementary information

**Fig. S1:** Identification of long non-coding RNAs (lncRNAs) and mRNAsI involved in high altitude hypoxia adaptation in liver tissue. Coding potentiality filter using Coding Potential Calculator, Pfam, Phylogenetic codon substitution frequency, and Coding-Non-Coding Index (A). The boxplot shows the expression levels (log10 (FPKM +1)) of lncRNAs and mRNAs (B). Transcript lengths distribution of mRNAs (C) and lncRNAs (D). Exon number distribution of mRNAs (E) and lncRNAs (F). Open reading frame (ORF) length distribution of mRNAs (G) and lncRNAs (H).

**Fig. S2:** The correlation coefficient of all liver and lung samples. The correlation coefficient tends toward 1 between samples from the same group indicating that it is reasonable to perform further data analyses

**Fig. S3:** The circus plot of the chromosomal distribution of DE lncRNAs and mRNAs in (A) liver and (B) lung tissue. The outermost ring represents different chromosomes. From outer ring to inner ring in order as mRNA (AW vs HS), lncRNA (AW vs HS), mRNA (HB vs HS), lncRNA (HB vs HS), mRNA (GJ vs HS), lncRNA (GJ vs HS), mRNA (WT vs HS), lncRNA (WT vs HS). 1< log2FC <5 (orange), 5< log2FC <Inf (yellow), log2FC =Inf (red), -5< log2FC <-1 (purple), -Inf< log2FC <5 (blue), log2FC =-Inf (green).

**Fig. S4:** Interaction network of DEGs in five sheep populations from different altitude. (A) mRNA–mRNA interaction network of DE mRNAs in liver tissue. (B) mRNA–mRNA interaction network of DE mRNAs in lung tissue. (C) lncRNA–mRNA interaction network in liver tissue. (D) lncRNA–mRNA interaction network in lung tissue. Circles and squares represent mRNAs and lncRNAs, respectively.

**Table S1:** The primers for mRNAs and lncRNAs.

**Table S2:** The determination results of the physiological and biochemical indices of five sheep populations.

**Table S3:** Statistics of clean reads of liver and lung tissue samples from five sheep populations.

**Table S4:** Identification of lncRNAs and mRNAs in sheep liver and lung tissue.

**Table S5:** The DE mRNAs and lncRNAs in all groups by pairwise comparison.

**Table S6:** The DE mRNAs and lncRNAs of the pairwise comparison between Tibetan sheep and Hu sheep (AW vs HS, HB vs HS, GJ vs HS, WT vs HS).

**Table S7:** Annotation of DE mRNAs and the targeted genes of DE lncRNAs.

**Table S8:** Significantly enriched GO terms and KEGG pathways of targeted genes of DE lncRNAs.

**Table S9:** The top 10 DE mRNAs with the highest degree and lncRNAs that interacted with more target genes.

**Table S10:** qRT-PCR verification of 7 DE lncRNAs, 13 target genes, and 20 DE mRNAs.
