# Supplementary figures and images for: Comparative analysis of long noncoding RNA and mRNA expression provides insights into adaptation to hypoxia in Tibetan sheep
Source: Sci Rep. 2022 Apr 21;12:6597. doi: 10.1038/s41598-022-08625-y (PMC9023463; doi:10.1038/s41598-022-08625-y)

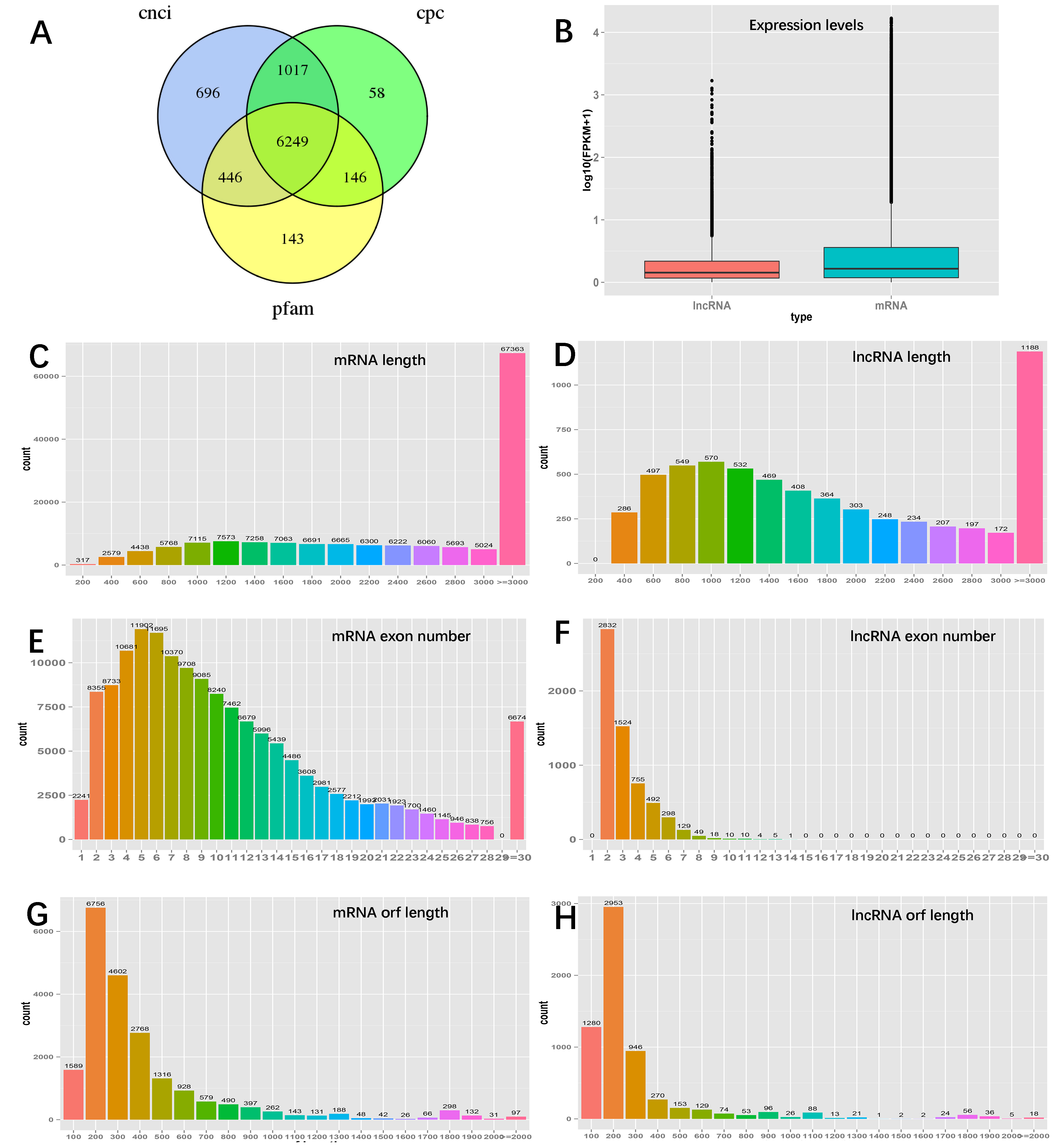

Supplement: Supplementary file 2 — Supplementary Figure S1. [file 41598_2022_8625_MOESM2_ESM.tif]

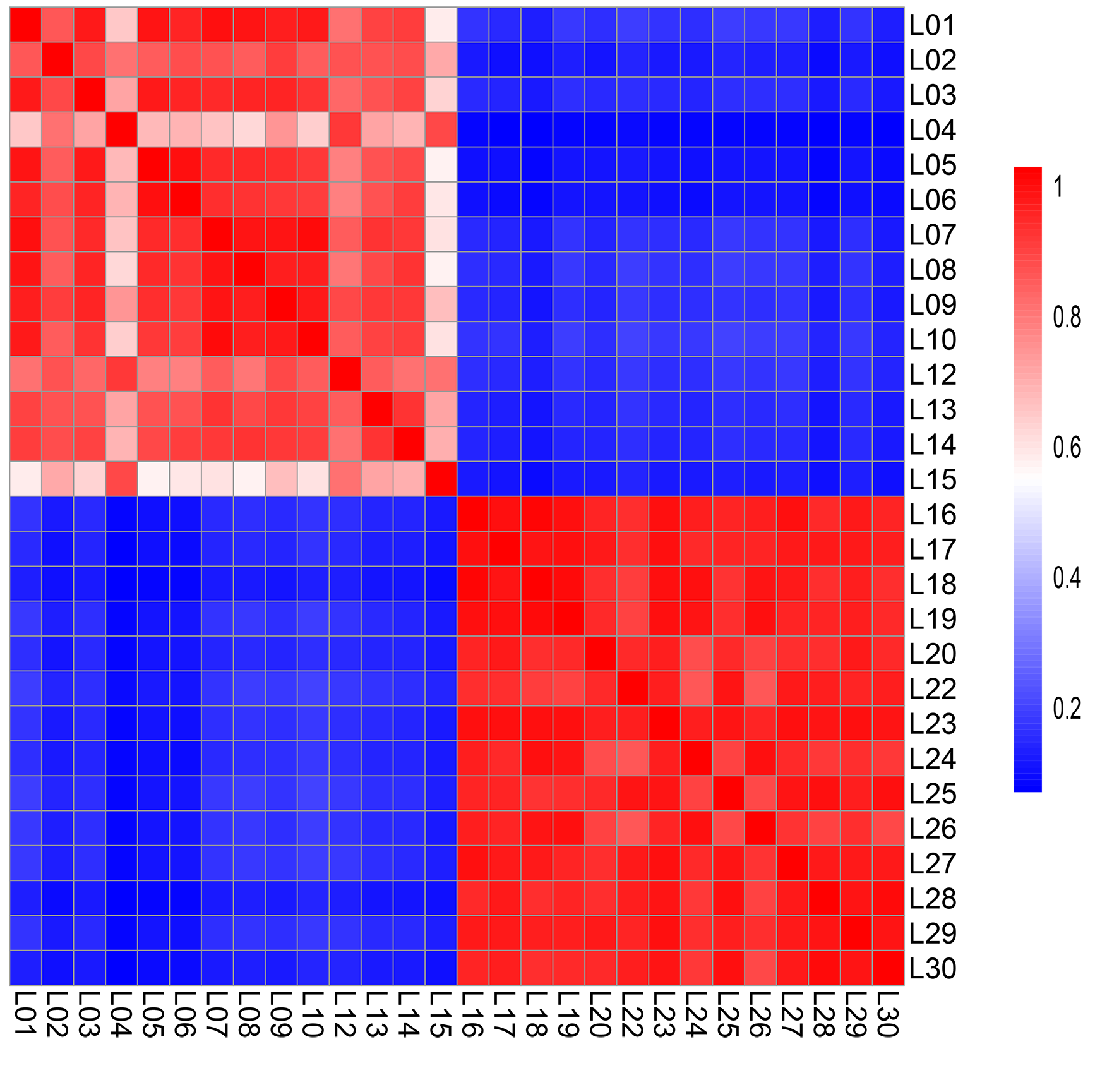

Supplement: Supplementary file 3 — Supplementary Figure S2. [file 41598_2022_8625_MOESM3_ESM.tif]

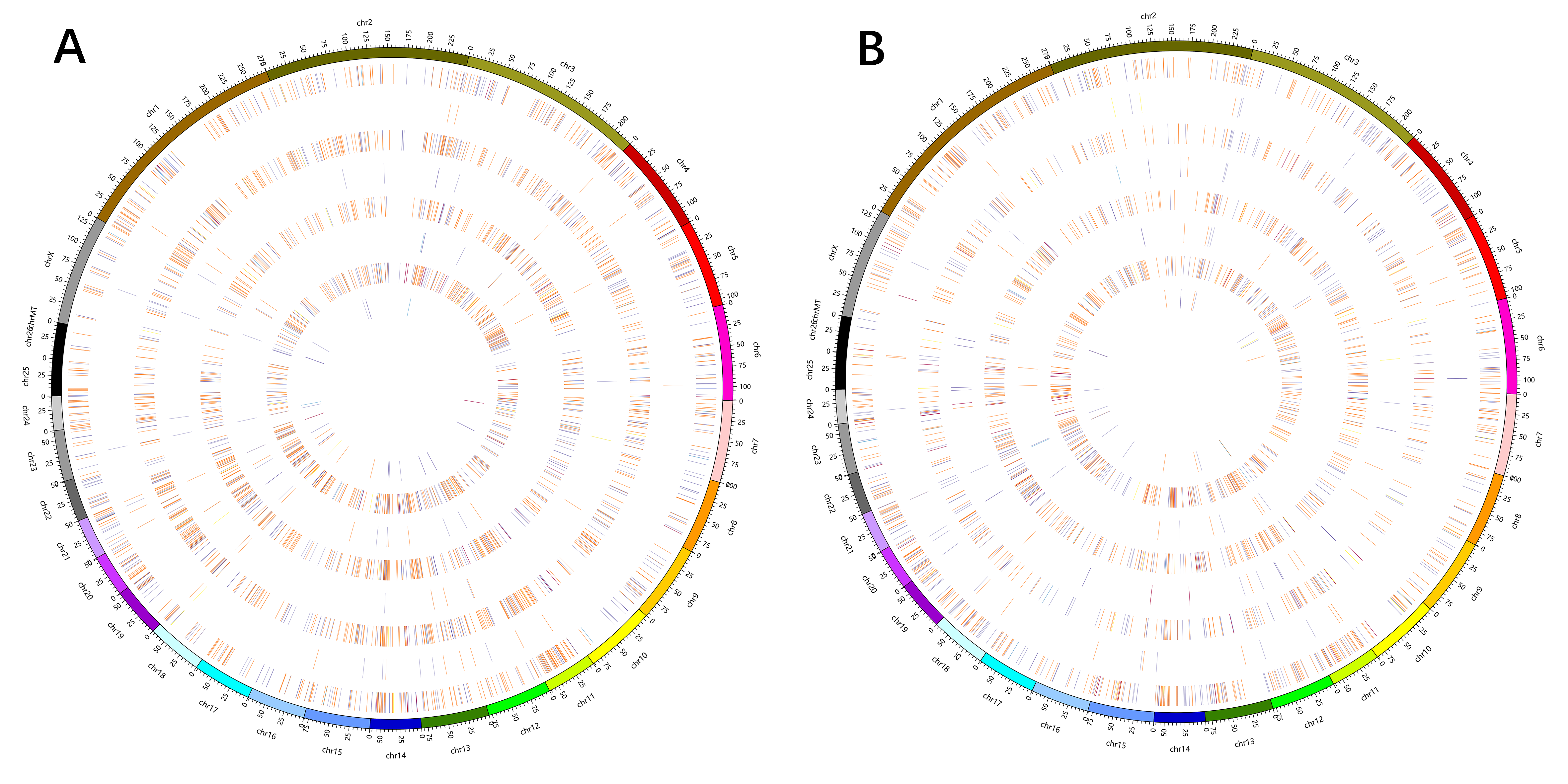

Supplement: Supplementary file 4 — Supplementary Figure S3. [file 41598_2022_8625_MOESM4_ESM.tif]

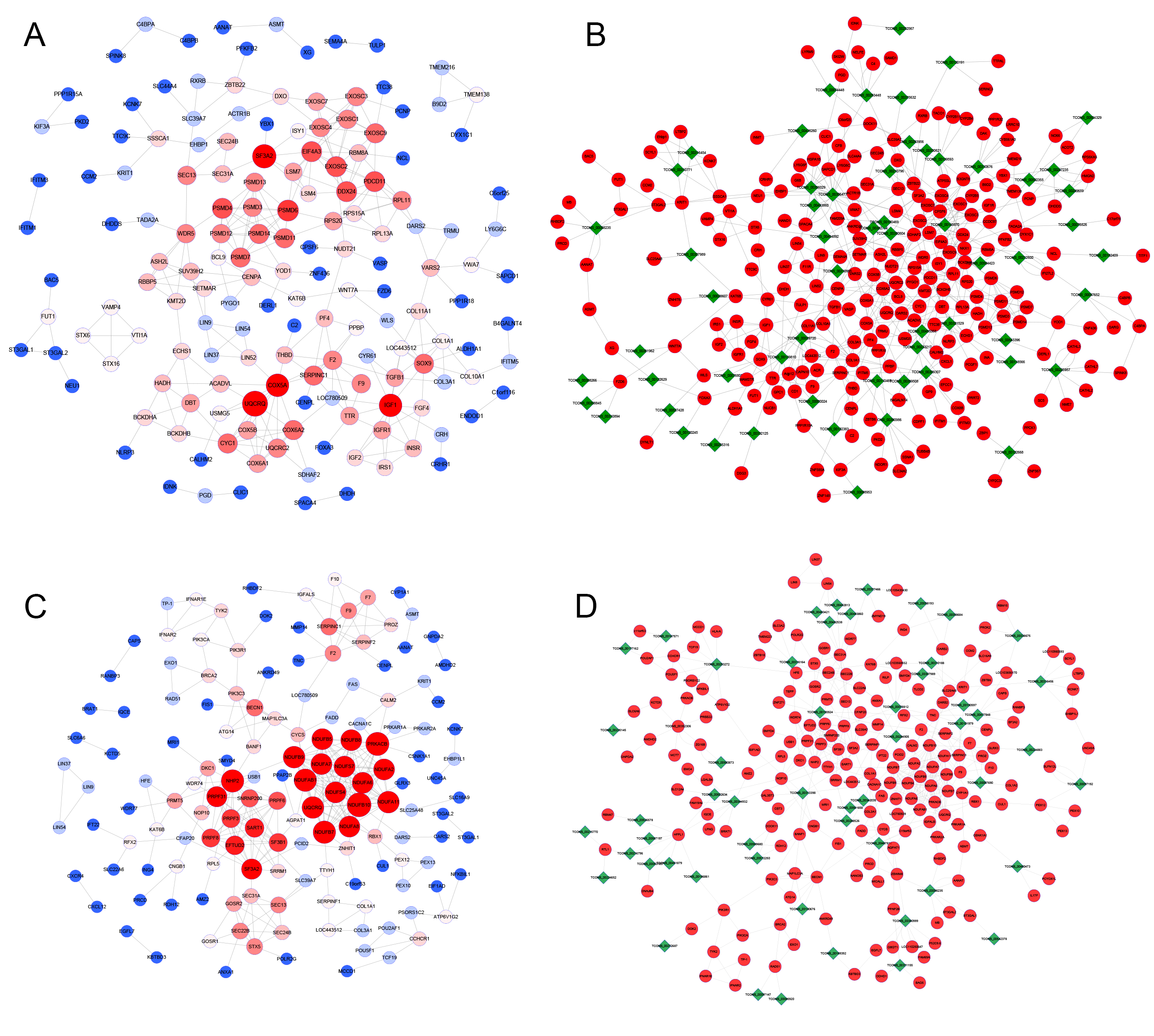

Supplement: Supplementary file 5 — Supplementary Figure S4. [file 41598_2022_8625_MOESM5_ESM.tif]
